# Supplementary material for: MetaRibo-Seq measures translation in microbiomes
Source: Nat Commun. 2020 Jun 29;11:3268. doi: 10.1038/s41467-020-17081-z (PMC7324362; doi:10.1038/s41467-020-17081-z)
Supplement: Supplementary file 10 — Supplementary Data 7 [file 41467_2020_17081_MOESM10_ESM.zip › File2/Confidence_High_Taxonomy/333910_out.krona.html]

Javascript must be enabled to view this page.

members
magnitude
magnitudeUnassigned
count
unassigned
taxon
rank

333910\_out

35

superkingdom
35
2

35
1239
phylum

1897036
4

SRS017916\_contig\_number\_16875SRS019808\_contig\_number\_18065SRS1041038\_contig\_number\_15599SRS147139\_contig\_number\_33905
species

class
186801
25

order
25
186802

family
186803
8

8
1898203
species

SRS015782\_contig\_number\_42597SRS017191\_contig\_number\_contig-100\_1528.125845SRS019068\_contig\_number\_114240SRS024435\_contig\_number\_13627SRS045645\_contig\_number\_29331SRS047044\_contig\_number\_19037SRS064757\_contig\_number\_contig-100\_395.137866SRS1055022\_contig\_number\_22698

31979
17
family

1485
17
genus

59620
17

SRS013476\_contig\_number\_contig-100\_400.225490SRS015065\_contig\_number\_7248SRS017307\_contig\_number\_contig-100\_334.176670SRS019445\_contig\_number\_4049SRS020869\_contig\_number\_12185SRS024492\_contig\_number\_19436SRS042628\_contig\_number\_36664SRS053398\_contig\_number\_25569SRS054352\_contig\_number\_contig-100\_1096.135722SRS063985\_contig\_number\_contig-100\_271.162674SRS075821\_contig\_number\_contig-100\_1073.135339SRS078419\_contig\_number\_12513SRS098571\_contig\_number\_31350SRS1041133\_contig\_number\_24348SRS144506\_contig\_number\_57090SRS149784\_contig\_number\_26042SRS893270\_contig\_number\_23526
species

species

SRS055982\_contig\_number\_3270SRS077127\_contig\_number\_7334SRS098881\_contig\_number\_24976SRS1041136\_contig\_number\_22839SRS142781\_contig\_number\_9667SRS144714\_contig\_number\_28956
6
2292887
